# Hallux valgus interphalangeus is more common in juvenile-onset hallux valgus than in adult-onset hallux valgus

**DOI:** 10.1186/s13018-024-05408-1

**Published:** 2024-12-26

**Authors:** An Seong Chang, Sei Wook Son, Ppuri Park, Hak Jun Kim, Sang Hyeon Hwang, Sang Geon Park, Young Hwan Park

**Affiliations:** 1 Department of Orthopaedic Surgery, Korea University Guro Hospital, 148 Gurodong-ro, Guro- gu, Seoul, 08308 Korea; 2 Department of Mechatronics Convergence, Changwon National University, Changwon, Korea

**Keywords:** Great toe, Foot deformities, Age of onset, Radiography

## Abstract

**Background:**

This study aimed to compare the prevalence of hallux valgus interphalangeus (HVI) in juvenile-onset hallux valgus and adult-onset hallux valgus and to analyze the correlation between the hallux interphalangeal angle (HIA) and other radiographic parameters in juvenile-onset hallux valgus.

**Methods:**

This retrospective study included 640 feet and 320 patients with hallux valgus (160 juvenile-onset and 160 adult-onset cases). Eight radiographic parameters were measured: HIA, hallux valgus angle, intermetatarsal angle, talonavicular coverage angle, anteroposterior talocalcaneal angle, lateral talocalcaneal angle, lateral talo-first metatarsal angle, and calcaneal pitch. The two groups were compared based on the radiographic parameters, and the correlation between the HIA and other radiographic parameters in juvenile-onset valgus was analyzed.

**Results:**

The prevalence of HVI in juvenile-onset hallux valgus (63%) was higher than that in adult-onset hallux valgus (28%), and juvenile-onset hallux valgus demonstrated a greater HIA than that displayed by adult-onset hallux valgus (mean ± standard deviation, 12.9 ± 5.7 and 8.3 ± 5.2, respectively). In juvenile-onset valgus, the HIA was negatively correlated with the hallux valgus (*r* = -0.218, *p* < 0.001) and intermetatarsal angles (*r* = -0.143, *p* = 0.015) and positively correlated with the talonavicular coverage (*r* = 0.240, *p* < 0.001) and anteroposterior talocalcaneal angles (*r* = 0.127, *p* = 0.008).

**Conclusions:**

Juvenile-onset hallux valgus is associated with more HVI than that in adults. Moreover, forefoot abduction deformity is related to the progression of HVI. These findings highlight the need to consider concomitant HVI when juvenile-onset valgus is encountered.

**Level of evidence:**

Level III, retrospective comparative study.

## Introduction

Hallux valgus interphalangeus (HVI) is characterized by the lateral deviation of the great toe toward the other toes and is defined by an increase in the hallux interphalangeal angle (HIA) of greater than 10° [1, 2]. The cause of HVI is not yet known but is thought to be multifactorial, including growth plate abnormalities and external pressure [3–5]. Although HVI rarely causes symptoms, the condition poses a cosmetic concern.

HVI and hallux valgus are believed to be related to their proximity to the anatomical location and commonality of lateral deviations. However, several studies have demonstrated that HVI is more common in normal feet than in hallux valgus and is negatively correlated with the severity of hallux valgus [6–8]. Nevertheless, surgeons remain cautious in cases where hallux valgus accompanies HVI, as HVI significantly influences surgical techniques for hallux valgus correction (e.g., Akin osteotomy) [9–11].

**Fig. 1:**
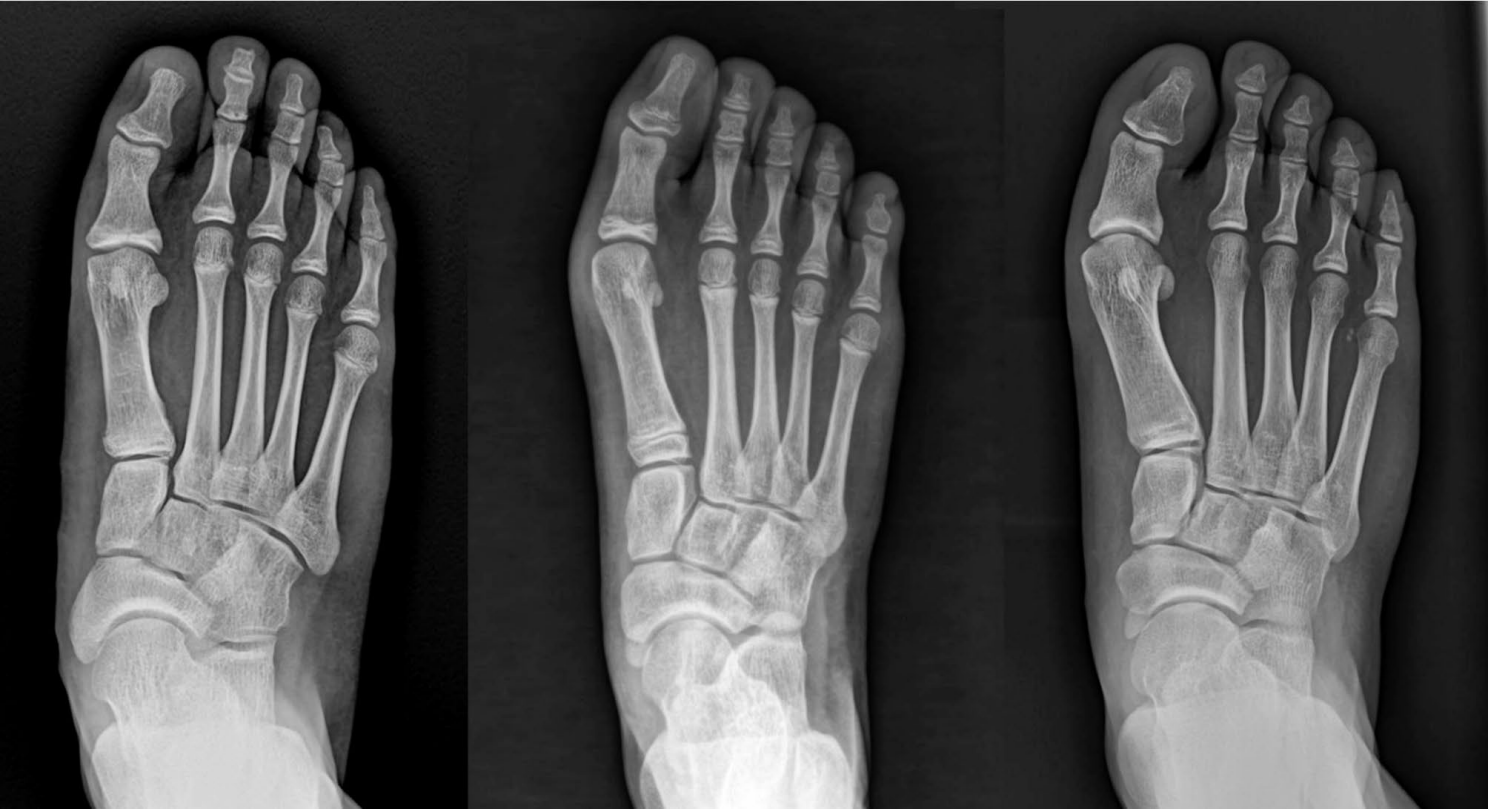
Standing anteroposterior foot radiographs of the patients with juvenile-onset hallux valgus with concomitant hallux valgus interphalangeus

When considering the full age spectrum of hallux valgus, our anecdotal experience demonstrated that HVI is more often associated with juvenile-onset than with adult-onset hallux valgus (Fig. 1). Furthermore, HVI seems to be prominent in juvenile-onset hallux valgus when accompanied by flatfoot. Age-related differences in the presence of HVI are important in diagnosing and treating hallux valgus according to onset time. However, no studies have identified differences in the association between HVI and hallux valgus according to onset time; therefore, confirming our empirical questions in the literature is difficult. To address this issue, this study aimed to (1) compare the radiographic features of juvenile-onset hallux valgus and adult-onset hallux valgus and (2) analyze the correlation between HIA and other radiographic parameters in juvenile-onset hallux valgus. We hypothesized that HVI would be more common in juvenile-onset hallux valgus than in adult-onset hallux valgus and would be affected by the accompanying foot deformity.

## Materials and methods

After obtaining approval from the local Ethics Committee, an electronic medical chart search was performed for patients with hallux valgus (International Classification of Diseases 10th Revision code, M 20.1) who visited our institution between March 2016 and May 2024. During the study period, hallux valgus was diagnosed when patients exhibited a hallux valgus angle (HVA) greater than 20° and an intermetatarsal angle (IMA) greater than 10° on bilateral standing weight-bearing radiographs of the feet. Patients with unilateral hallux valgus, congenital anomalies, neuromuscular diseases, or a history of foot trauma affecting the foot appearance were excluded. Juvenile-onset hallux valgus was defined as the deformity onset at ≤ 16 years of age, with the presence of open physes in the feet. Adult-onset hallux valgus was defined as deformity onset at > 16 years of age, with the presence of closed foot physes. Patients with adult-onset hallux valgus who were unaware of the onset time of their deformity were also excluded.

Once these inclusion and exclusion criteria were filtered, 1796 patients were identified as having hallux valgus (160 juvenile-onset and 788 adult-onset). To compare juvenile-onset and adult-onset valgus, we decided to perform a 1:1 matching analysis. A strict matching process incorporating multiple variables was employed to ensure similarities in the deformity severity and patient demographics between the two patients in each pair. All matching was performed by one of the authors (ASC) who did not participate in the radiographic measurements or result analysis. Matching criteria included sex, HVA, and IMA. Following the matching process, 160 cases (320 feet) of juvenile-onset hallux valgus and 160 (320 feet) of adult-onset hallux valgus were included in this study.

### Radiographic measurements

**Fig. 2:**
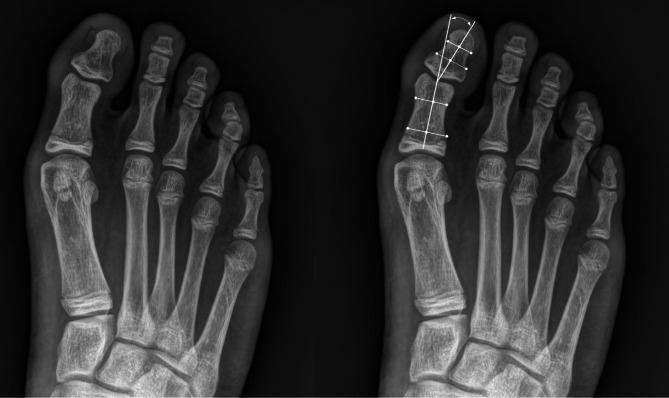
Radiologic measurement assessing the degree of hallux valgus interphalangeus. The hallux interphalangeal angle is the angle formed by the longitudinal axes of the proximal and distal phalanx of the first toe

Two authors (SWS and YHP) independently performed all radiographic measurements using a Picture Archiving and Communication System (PiViewSTAR; Infinitt, Seoul, Republic of Korea), and the means of the two measurements were used for statistical analysis. The radiographic parameters were defined as follows: HVA, the angle formed by a longitudinal axis of the proximal phalanx and that of the first metatarsal bone; IMA, the angle formed by a longitudinal axis of the first metatarsal bone and a longitudinal axis of the second metatarsal bone; HIA, the angle formed by longitudinal axes of the proximal and distal phalanx of the first toe (Fig. 2); talonavicular coverage angle (TNCA), the angle formed by a line that joins the medial and lateral articular margins of the talus, and a line between that joins the medial and lateral articular margins of the navicular. Furthermore, the anteroposterior talocalcaneal angle (AP-TCA), was defined as the angle formed by a longitudinal axis of the talus and a longitudinal axis of the calcaneus on an anteroposterior radiograph; lateral talocalcaneal angle (Lat-TCA), the angle formed by a line drawn along the lower margin of the calcaneus and a longitudinal axis of the talus on a lateral radiograph; lateral talo-first metatarsal angle (Lat-TMA), the angle between the long axis of talar head and the long axis of first metatarsal bone; calcaneal pitch (CP), the angle formed by a line drawn along the edge of the plantar soft tissue shadow and a line drawn along the lower calcaneal margin [2, 6, 12, 13].

### Statistical analysis

The Kolmogorov–Smirnov test was employed to determine data normality. An independent t-test was used to compare continuous variables, including the radiographic parameters of juvenile- and adult-onset hallux valgus. The chi-squared test was used to compare categorical variables. A linear regression model was used to investigate the relationship between HIA and other radiographic parameters of juvenile-onset hallux valgus. All statistical analyses were performed using SPSS (version 23.0; IBM Corp., Armonk, NY, USA), with statistical significance set at *p* < 0.05.

## Results

The mean age was 12.7 years (range, 7–16 years) in the juvenile-onset hallux valgus and 56.0 years (range, 31–73 years) in the non-juvenile-onset hallux valgus group. In the juvenile-onset hallux valgus group, 31 patients were male and 129 were female; this ratio was the same as that in the adult-onset hallux valgus group.

In the comparison of radiographic parameters, HIA was the only parameter that displayed intergroup differences; juvenile-onset hallux valgus exhibited a greater HIA than that displayed by adult-onset hallux valgus (Table 1). The prevalence of HVI in juvenile-onset hallux valgus (63%) was higher than that in adult-onset hallux valgus (28%).

**Table 1:** Comparison of radiographic parameters between the juvenile and adult-onset hallux valgus

	Juvenile-onset hallux valgus	Adult-onset hallux valgus	*p*-value
HVA	23.1 ± 10.7	24.4 ± 11.7	0.172
IMA	10.9 ± 3.2	11.3 ± 3.6	0.228
HIA	12.9 ± 5.7	8.3 ± 5.2	**< 0.001**
TNCA	20.1 ± 9.3	21.7 ± 10.7	0.415
AP-TCA	26.3 ± 9.9	25.8 ± 8.5	0.175
Lat-TCA	44.1 ± 10.1	44.8 ± 9.6	0.215
Lat-TMA	7.6 ± 7.0	8.3 ± 5.9	0.208
CP	18.6 ± 8.9	17.3 ± 9.1	0.349

Data are presented as mean ± standard deviation. The results in bold font are considered statistically significant (*p* < 0.05)

HVA, hallux valgus angle; IMA, intermetatarsal angle; HIA, hallux interphalangeal angle; TNCA, talonavicular coverage angle; AP-TCA, anteroposterior talocalcaneal angle; Lat-TCA, lateral talocalcaneal angle; Lat-TMA, lateral talo-first metatarsal angle; CP, calcaneal pitch

When analyzing the correlations among the radiographic parameters in juvenile-onset hallux valgus, the HVA, IMA, TNCA, and AP-TCA were correlated with HIA (Table 2). The HIA value tended to decrease as the HVA and IMA values increased, meanwhile, the HIA value tended to increase as the TNCA and AP-TCA values increased.

**Table 2:** Correlations among the radiographic parameters in juvenile-onset hallux valgus

	HVA	IMA	HIA	TNCA	AP-TCA	Lat-TCA	Lat-TMA	CP
HVA	1.000	0.376^**^	-0.218^**^	0.024	0.011	-0.003	0.091	-0.073
IMA		1.000	-0.143^*^	0.171^**^	0.113	-0.041	0.164^**^	-0.192^**^
HIA			1.000	0.240^**^	0.127^**^	0.054	0.001	0.157
TNCA				1.000	0.202^**^	0.113	0.255^**^	-0.356^**^
AP-TCA					1.000	0.070	-0.042	0.008
Lat-TCA						1.000	0.399^**^	0.307^**^
Lat-TMA							1.000	-0.258^**^
CP								1.000

HVA, hallux valgus angle; IMA, intermetatarsal angle; HIA, hallux interphalangeal angle; TNCA, talonavicular coverage angle; AP-TCA, anteroposterior talocalcaneal angle; Lat-TCA, lateral talocalcaneal angle; Lat-TMA, lateral talo-first metatarsal angle; CP, calcaneal pitch

*Correlation is significant at the 0.05 level (two-tailed)

**Correlation is significant at the 0.01 level (two-tailed)

## Discussion

To verify our hypothesis that HVI would be more common in juvenile-onset hallux valgus than in adult-onset hallux valgus and would be affected by accompanying foot deformities, we analyzed the radiographic features of juvenile- and adult-onset hallux valgus. The primary finding of our study was that the prevalence of HVI was higher in juvenile-onset than in adult-onset hallux valgus. Furthermore, in juvenile-onset hallux valgus, the HIA was significantly correlated with the TNCA and AP-TCA, indicating that HVI was affected by forefoot abduction. Based on this study, we suggest that clinicians should be aware of concomitant HVI when diagnosing and treating juvenile-onset hallux valgus, particularly in patients with forefoot abduction.

### Prevalence and significance of HVI in juvenile-onset hallux valgus

Kim et al. discovered that 20% of patients with hallux valgus had concomitant HVI in their radiographic review, however, they did not stratify the prevalence based on the age of onset [8]. The results of this study revealed a difference in the prevalence of HVI between juvenile-onset and adult-onset hallux valgus. In juvenile-onset hallux valgus, HVI was identified in 63% of cases, whereas in adult-onset hallux valgus, the condition was diagnosed in only 28% of cases. This finding supports the notion that HVI is relatively common in young individuals with hallux valgus, suggesting that developmental or biomechanical factors may contribute to this association. The high prevalence in juveniles may be attributed to the ongoing growth and development of the foot, where abnormal forces and pressures may influence the interphalangeal joint [14].

### Radiographic differences and implications

Regarding radiographic parameters, this study discovered that HIA was significantly greater in juvenile-onset hallux valgus than in adult-onset hallux valgus. This difference highlights the distinct morphological characteristics of juvenile-onset hallux valgus and emphasizes the need for age-specific diagnostic and therapeutic approaches. Surgeons should consider the presence of HVI when planning corrective procedures for juvenile patients, as this could affect the choice and outcome of surgical interventions, including Akin osteotomy.

### Correlation with foot deformity parameters

In this study, the value of HIA tended to decrease as the values of HVA and IMA increased, similar to what was established in previous studies [6–8]. In the growth period, excessive pressure on the forefoot caused by footwear can lead to lateral underdevelopment of the distal phalangeal joint, resulting in an increased HIA. However, in patients with increased HVA, the lateral deformity of the proximal phalanx reduces the pressure exerted on the distal phalangeal joint, potentially mitigating the progression of the HIA [15]. Meanwhile, the analysis further revealed that in juvenile-onset hallux valgus, HIA was positively correlated with TNCA and AP-TCA, which has not been reported previously. These parameters are indicative of foot deformity and suggest a link between forefoot abduction and increased HIA in juvenile patients. Forefoot abduction could exacerbate the lateral deviation of the hallux interphalangeal joint due to altered biomechanics and weight distribution across the foot [16, 17]. This correlation underscores the importance of evaluating and potentially addressing foot deformity in the management of juvenile-onset hallux valgus to prevent or mitigate HVI. The other parameters, Lat-TCA and CP, did not exhibit a significant correlation, implying that HVI is unrelated to the hindfoot malalignment observed in the flatfoot.

### Clinical relevance and future research

The strength of this study is that, unlike previous studies, this is the first to investigate the association between HVI and hallux valgus according to the age of onset. Our findings have several clinical implications. First, the high prevalence of HVI in juvenile-onset hallux valgus necessitates a thorough radiographic assessment of young patients presenting with hallux valgus, as this can influence both conservative and surgical treatment strategies. Second, the association between forefoot deformities and HIA in juvenile patients suggests that interventions aimed at correcting forefoot abduction could potentially reduce the severity of HVI.

Future research should focus on longitudinal studies to track the progression of HVI and hallux valgus from childhood to adulthood and provide further insights into the natural history and optimal management of these deformities. In addition, exploring the underlying biomechanical and genetic factors that contribute to the development of HVI in juveniles could lead to the development of targeted prevention and treatment strategies.

### Limitations

This study has two limitations. First, concerns were present about grouping patients as the onset of deformity could be misreported. If the deformity was subtle when the patients were young and noticed as adults, an error could be made in classifying the patient as having adult-onset hallux valgus. To minimize bias, we excluded cases with unclear onset times from medical records during the study design stage. Second, this study could not utilize the calcaneal axis parameter to analyze the correlation between HVI and foot deformity. This was due to missing values, as the calcaneal axial view was not obtained for all patients during the study period. Therefore, we believe that further studies that include heel alignment analysis are needed to elucidate an association between the HVI and flatfoot.

## Conclusion

This study demonstrated that HVI is more prevalent and severe in juvenile-onset hallux valgus than in adult-onset hallux valgus. The significant correlation between HIA and forefoot abduction-related parameters in juveniles highlights the need for a comprehensive assessment and treatment of combined deformities in managing juvenile-onset hallux valgus. These findings emphasize the importance of age-specific diagnostic and therapeutic approaches to improve the outcomes of patients with hallux valgus and HVI.

## Data Availability

No datasets were generated or analysed during the current study.
